# Examination of the Ligand-Binding and Enzymatic Properties of a Bilin-Binding Protein from the Poisonous Caterpillar *Lonomia obliqua*

**DOI:** 10.1371/journal.pone.0095424

**Published:** 2014-06-27

**Authors:** Ana B. G. Veiga, José M. C. Ribeiro, Ivo M. B. Francischetti, Xueqing Xu, Jorge A. Guimarães, John F. Andersen

**Affiliations:** 1 Laboratory of Malaria and Vector Research, National Institute of Allergy and Infectious Disease, National Institutes of Health, Rockville, Maryland, United States of America; 2 Center of Biotechnology, Universidade Federal do Rio Grande do Sul, Porto Alegre RS, Brazil; Russian Academy of Sciences, Institute for Biological Instrumentation, Russian Federation

## Abstract

The bilin-binding proteins (BBP) from lepidopteran insects are members of the lipocalin family of proteins and play a special role in pigmentation through the binding of biliverdin IXγ. Lopap, a BBP-like protein from the venom of the toxic caterpillar *Lonomia obliqua* has been reported to act as a serine protease that activates the coagulation proenzyme prothrombin. Here we show that BBPLo, a variant of lopap from the same organism binds biliverdin IXγ, forming a complex that is spectrally identical with previously described BBP proteins. Although BBPLo is nearly identical in sequence to lopap, no prothrombinase activity was detected in our recombinant preparations using reconstituted systems containing coagulation factors Xa and Va, as well as anionic phospholipids. In addition to biliverdin, BBPLo was found to form a 1∶1 complex with heme prompting us to examine whether the unusual biliverdin IXγ ligand of BBPs forms as a result of oxidation of bound heme in situ rather than by a conventional heme oxygenase. Using ascorbate or a NADPH^+^-ferredoxin reductase-ferredoxin system as a source of reducing equivalents, spectral changes are seen that suggest an initial reduction of heme to the Fe(II) state and formation of an oxyferrous complex. The complex then disappears and a product identified as a 5-coordinate carbonyl complex of verdoheme, an intermediate in the biosynthesis of biliverdin, is formed. However, further reaction to form biliverdin was not observed, making it unlikely that biliverdin IXγ is formed by this pathway.

## Introduction

Previous studies have shown that cuticular bristles of the Brazilian caterpillar *Lonomia obliqua* produce a potent venom that causes severe bleeding reactions and renal failure after contact with human skin. One of the functionally important components of the venom is a prothombin activator. Reis et al. [1] fractionated extracts of the caterpillar integument by chromatographic procedures and identified the prothrombin activator as a member of the lipocalin protein family which they gave the name lopap. Recombinant lopap was produced by bacterial expression and tested in enzymatic assays against prothrombin and factor X. The recombinant protein was found to weakly activate prothrombin but not factor X. The activity was consistent with a serine protease in that it required calcium ions and was inhibited by PMSF. It was proposed, based on molecular modeling, that lopap exists as a tetrameric protein having one catalytic site per monomer. This remarkable finding would bestow a new functional activity on the lipocalin family of proteins which normally bind small molecule ligands or other proteins.

**Figure 1:**
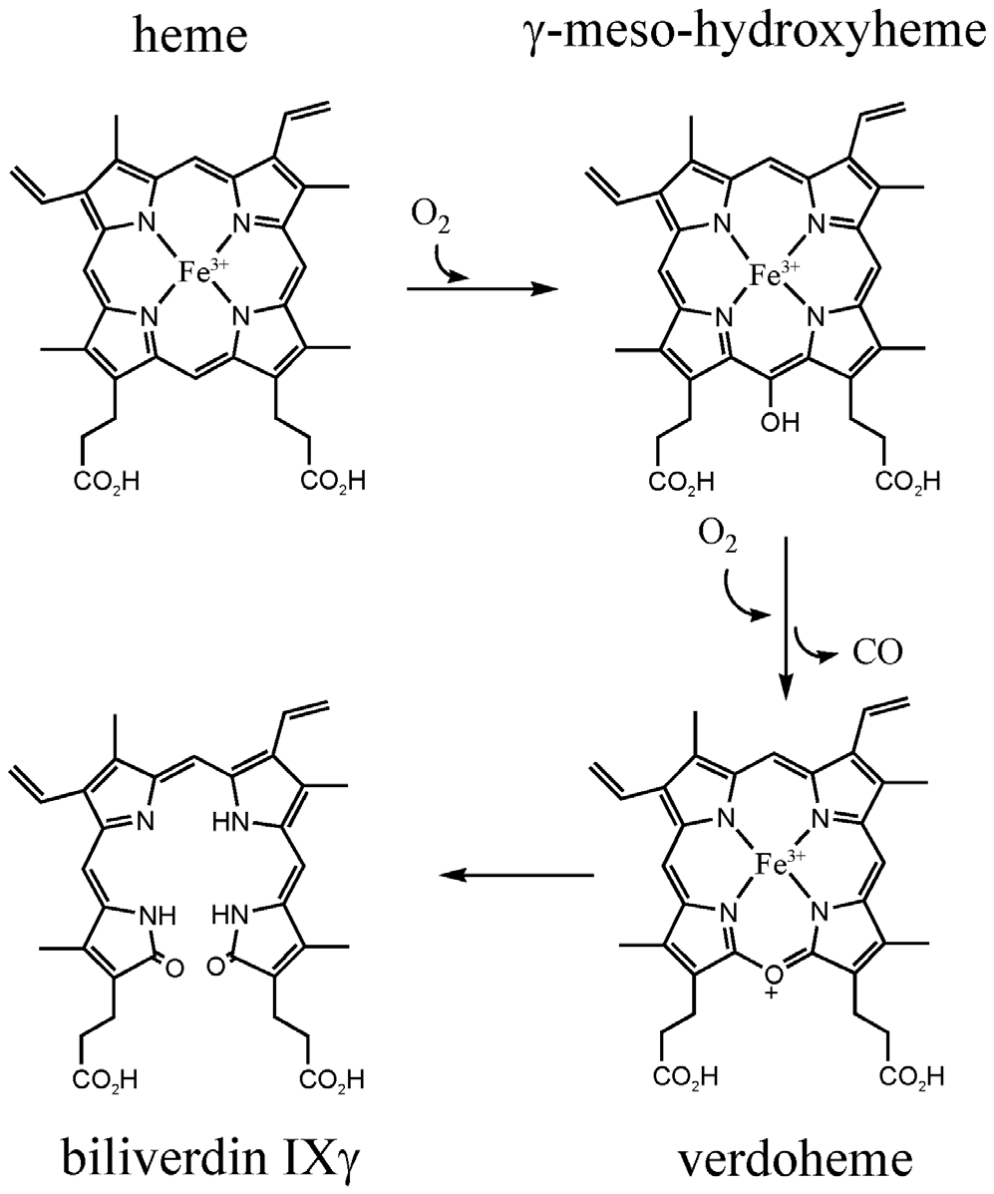
Proposed reaction pathway for the formation of biliverdin IXγ from heme by a heme oxygenase-like mechanism.

Based on sequence similarity, lopap belongs to the biliverdin-binding protein (BBP) family. Members of this group are present at high concentration in the epidermis and integument of many lepidopteran insect species, where they are thought to provide UV-protectant pigmentation [2], [3]. In all organisms studied to date, biliverdin is produced from heme by the action of heme oxygenase with concomitant release of one molecule of carbon monoxide per molecule of heme. Mammalian heme oxygenases produce only the α isomer of biliverdin IX [4], while a bacterial form of the enzyme from *Pseudomonas aeruginosa* produces a mixture of the β and δ isomers [5]. The lepidopteran BBPs contain only biliverdin IXγ, which is not a product of any known heme oxygenase enzyme, suggesting that the ligand may be produced by an unconventional mechanism (Fig. 1). A heme oxygenase form responsible for production of this ligand has not been identified.

In this study we describe the production BBPLo, a recombinant version of a variant of lopap from *L. obliqua*, and examine its ability to activate prothrombin [6], [7], [8]. Since it resembles a biliverdin binding protein, we have also examined its ligand binding properties. In our hands, recombinant BBPLo does not activate prothrombin as reported in studies with lopap. The protein does bind biliverdin-IXγ in a complex that is stable to size exclusion chromatography, indicating that it functions as a BBP. Surprisingly, we find that heme is also bound by BBPLo in an apparent 1∶1 complex. This observation prompted us to test the hypothesis that the unusual biliverdin IXγ ligand is formed by in situ oxidation of bound heme rather than by a conventional heme oxygenase. We show that bound heme is susceptible to coupled oxidation using ascorbate as an electron donor. However, a slow reaction rate, lack of complete conversion to biliverdin, and lack of absolute reaction regiospecificity suggest that an in situ oxidation of heme does not represent the normal physiological path to biliverdin IXγ and that another heme oxygenase must be present to accomplish this task.

## Materials and Methods

### Materials

Biliverdin IXα was purchased from Frontier Scientific Inc., Logan, UT, spinach ferredoxin-NADP^+^ reductase, spinach ferredoxin, NADP+, glucose-6-phosphate, and glucose-6-phosphate dehydrogenase were purchased from Sigma-Aldrich. Hemin was obtained from Fluka. *Manduca sexta* insecticyanin was a gift of Dr. J. Winzerling, University of Arizona. Biliverdin IXγ was obtained by extracting the insecticyanin sample with methanol and removing the precipitated protein by centrifugation. Human factor Xa, prothrombin and thrombin were obtained from Calbiochem. Factor Va was obtained from Haematologic Technologies, and S-2239 was obtained from Diapharma.

### Cloning and expression of BBPLo

The cDNA for BBPLo was obtained from a cDNA library of *L. obliqua* bristles [8] and modified to remove the signal peptide sequence. The cDNA was then cloned into the expression vector pET17b and expressed in *Escherichia coli* strain BL21(DE3)pLysS. Inclusion bodies obtained after induction by IPTG were washed in 20 mM Tris-HCl (pH 7.5), 150 mM NaCl, 1% Triton X-100, then suspended in 20 mM TrisHCl, pH 8.0, 6 M guanidine hydrochloride, 10 mM DTT. The recombinant protein was refolded by dilution into a large excess of 20 mM Tris (pH 8.5), 0.4 M arginine HCl and then concentrated by ultrafiltration. These refolding methods have been successfully used in numerous studies on insect lipocalin structure and function [9], [10].

Recombinant, refolded BBPLo was purified by gel filtration on Sephacryl S-100. The column was eluted at a flow rate of 1 ml/min with 50 mM Tris-HCl (pH 7.4), 150 mM NaCl and the fractions containing the purified protein were pooled and evaluated for purity and quantified by SDS-PAGE and UV-visible spectrophotometry. The protein was further purified by chromatography on Q-Sepharose using a gradient of 0–1 M NaCl in 20 mM Tris HCl pH 8.0 for elution.

### Binding of BBPLo to biliverdin IXα and to biliverdin IXγ

Recombinant BBPLo was incubated with either biliverdin IXα or biliverdin IXγ. Reactions were applied to a Superdex 75 HR 10/30 gel filtration HPLC column to analyze the binding of the pigments to recombinant BBPLo. The column was eluted at a flow rate of 0.3 ml/min with 50 mM Tris-HCl (pH 7.4), 150 mM NaCl, and the eluent was monitored using a Shimadzu SPD M10AV diode array detector.

### Preparation of a BBPLo-heme complex

Hemin, dissolved in DMSO, was added to a solution of 20 µM recombinant BBPLo in 20 mM Tris HCl pH 7.4, 150 mM NaCl to a final concentration of 50 µM. The complex was separated from unbound hemin by gel filtration chromatography on Sephacryl S-100 using 20 mM Tris HCl, pH 7.4, 150 mM NaCl as the elution buffer. Fractions were screened using UV-visible spectra and those containing the BBPLo-heme complex were pooled and concentrated using an Amicon Ultra 5,000 MWC filter.

### Determination of heme content of the complex

The specific content of heme in the BBPLo-heme complex was determined using the pyridine hemochrome method [11]. In a 1.0 ml cuvette, 300 µL, 100 µL 0.5 M NaOH, and 100 µL pyridine were added to 400 µL of the complex. A spectrum of the oxidized complex was then recorded. A small amount of sodium dithionite was then added and a spectrum of the reduced complex was recorded. The absorbance differences between 556 and 540 nm were then determined for each spectrum and the difference between the oxidized and reduced spectra were used to determine the heme content with an extinction coefficient of 23.98 mM^−1^ cm^−1^ [11].

### Isothermal titration calorimetry

Titrations were performed at 30°C in a Microcal VP-ITC microcalorimeter. After purification, recombinant BBPLo was dialyzed against 40 mM Tris HCl, pH 8.0. A heme solution, in the dialysis buffer, was prepared immediately before use by dilution of a 10 mM stock of hemin (in 100 mM sodium hydroxide) to a final concentration of 100 µM [12]. The calorimeter cell was filled with BBPLo solution at a concentration of 5 µM, and the syringe was filled with the 100 µM solution of hemin. At a stirring rate of 420 rpm, the hemin solution was injected into the BBPLo solution in 10 µL increments. The observed heats were converted to enthalpies and fit to a single-site binding model using the Microcal-Origin software package. The nitrophorin 2 (NP2) used in ITC experiments was produced in *E. coli* using methods described previously [9], [13], and purified by gel filtration chromatography on Sephacryl S-100.

### Reaction of the BBPLo-heme complex with sodium ascorbate

Sodium ascorbate was added to a concentration of 10 mM to a solution of the heme complex (9.3 µM in 20 mM Tris HCl, pH 7.4, 150 mM NaCl in a 1 mL cuvette). The reaction mixture was incubated at 37°C and UV-visible absorption spectra were recorded at various intervals.

### Reduction of the BBPLo-heme complex with the spinach ferredoxin-ferredoxin reductase system

Reducing equivalents were also provided using spinach ferredoxin-NADP^+^ reductase and spinach ferredoxin as an electron transfer system [14]. In this case 31 µg ferredoxin, 0.05 U ferredoxin-NADP^+^ reductase, 0.5 mM NADP^+^, and 2 mM glucose-6-phosphate were added to BBPLo-heme complex in a total volume of 0.8 mL 20 mM Tris HCl, pH 7.4, 150 mM NaCl. All components except the BBPLo-heme complex were also added to the reference cuvette. The cuvettes were incubated at 25°C in the spectrophotometer and an initial spectrum was taken. Glucose-6-phosphate dehydrogenase (2 U) was then added to both cuvettes to reduce the NADP^+^, and the reaction was monitored for 120 min. In some cases, reactions were allowed to incubate another 4–16 hr at room temperature.

### Extraction and detection of reduced pyridine hemochrome

To detect verdoheme as an intermediate, reactions were extracted after 2 hr with 10% (v/v) pyridine in chloroform, and absorbance spectra were recorded at room temperature.

### Analysis of reaction products

To analyze the reaction products obtained with ascorbate as the electron donor, the reaction mixture was concentrated in a 5000 MW cutoff filter (Amicon) and the buffer was replaced with water. The mixture was introduced directly into a Finnegan LCQ mass spectrometer.

To determine the reaction regioselectivity by HPLC and mass spectrometry, the reaction mixtures were acidified with 200 µL glacial acetic acid and 100 µL 5 M hydrochloric acid. The products were then extracted into 1 mL of chloroform which was evaporated to dryness under a stream of nitrogen [15]. The residue was taken up in 5% HCl-methanol and allowed to react overnight at 4°C. The resulting dimethyl esters were extracted into chloroform which was then evaporated to dryness. Prior to HPLC and mass spectral analysis, the dimethyl esters were dissolved in 40% acetonitrile in water.

The derivatized products were separated by HPLC on a 0.3×150 mm C-18 column using a gradient of 35–45% acetonitrile in water at a flow rate of 4 µl/min. The eluent first passed through a UV absorbance detector which was monitored at 310 nm, then to a Finnigan LCQ ion trap mass spectrometer.

### Tests for activation of prothrombin by BBPLo

Activation of prothrombin (Haematologic Technologies) by human factor Xa (FXa) in the presence of factor Va (FVa) and phospholipids was performed in TBS-Ca^2+^ (20 mM Tris-HCl, 150 mM NaCl, 5 mM CaCl_2_, 0.3% BSA, pH 7.5), using a discontinuous assay [16]. FXa (10 pM, final concentration), or BBPLo (1 or 10 µM) was incubated with human FVa in the presence of phosphatidylcholine/phosphatidylserine (PC/PS, 10 µM) for 5 min at 37°C. Reactions were initiated by addition of human prothrombin (1.4 µM). Aliquots of 25 µL were removed every 2 min into microplate wells containing 50 µL of TBS-EDTA (20 mM Tris-HCl, 150 mM NaCl, 20 mM EDTA, 0.1% BSA, pH 7.5) to stop reactions. After addition of 25 µL of chromogenic thrombin substrate S-2238 (312.5 µM), absorbance at 405 nm was recorded at 37°C for 15 min at 11-sec intervals using a Thermomax microplate reader (Molecular Devices, Menlo Park, CA) [17]. Initial velocities (mOD/min) obtained were used to calculate the amount of thrombin formed, using a standard curve [17]. Removal of any one of the components in the prothrombinase from the reaction resulted in a loss of activity.

Activation of prothrombin to thrombin by FXa alone was performed in 20 mM Tris/HCl, 150 mM NaCl, 5 mM CaCl_2_ and 0.3% BSA, pH 7.5, using a discontinuous assay as described [17]. FXa (10 nM final concentrations) or BBPLo (1 and 10 µM) were incubated with human prothrombin (1.4 µM), and aliquots of 25 µl were removed every 10 min into microplate wells containing 50 µl of 20 mM Tris/HCl, 150 mM NaCl, 20 mM EDTA and 0.3% BSA, pH 7.5. Thrombin formation was measured using the substrate S-2238 as described above.

## Results

### Cloning and expression of the BBPLo cDNA

**Figure 2:**
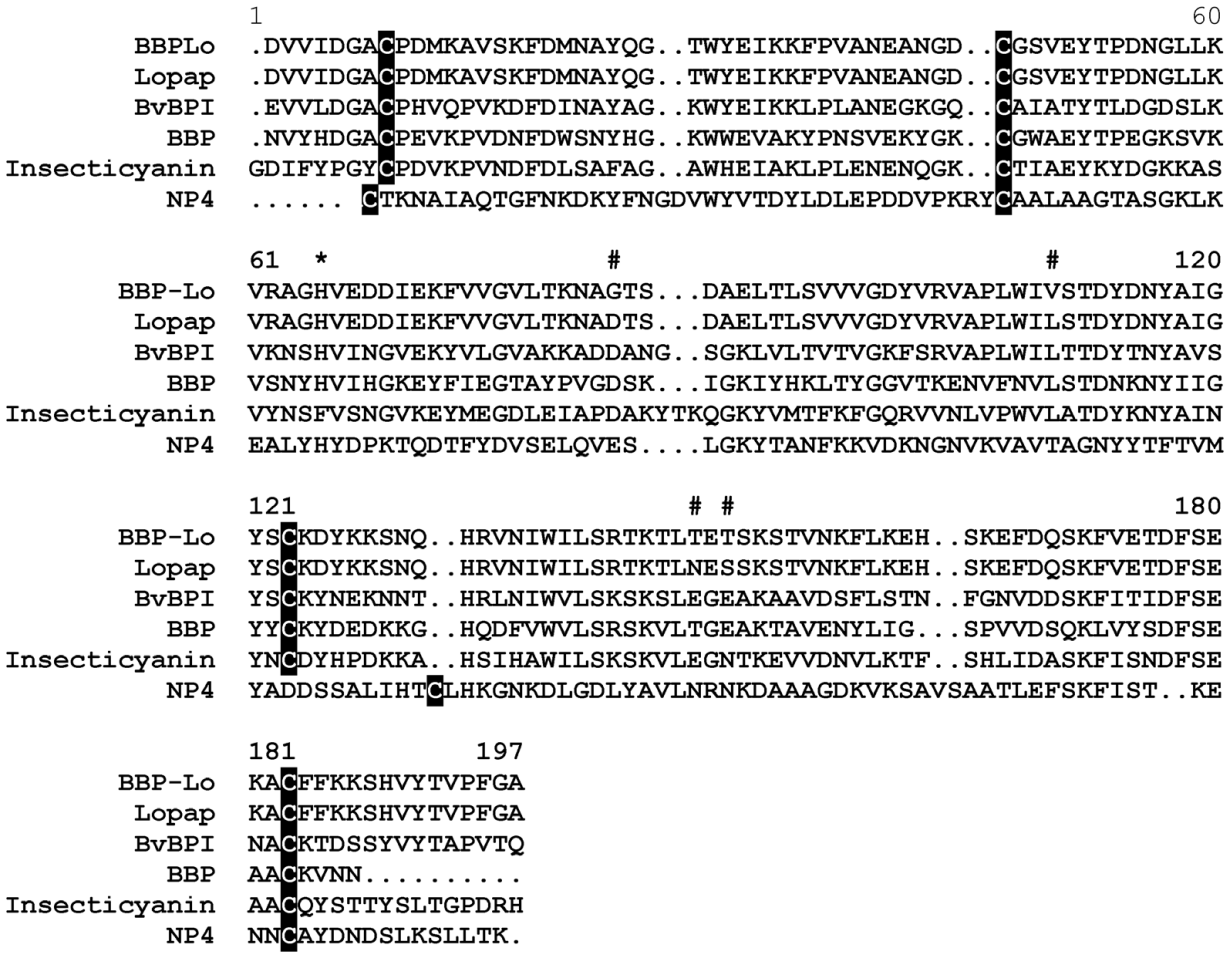
Alignment of *L. obliqua* BBPLo with lopap from *L. obliqua* (gi|59709575), biliverdin-binding protein I (BvBPI) from *Samia cynthia ricini* (gi|18857921), bilin-binding protein (BBP) from *Pieris brassicae* (gi|1705433), insecticyanin from *Manduca sexta* (gi|9716), and nitrophorin 4 (NP4) from *R. prolixus* (gi|3219833). Conserved cysteine residues are shaded in black, the binding pocket histidine residue is marked with an asterisk, and the amino acid positions differing between BBPLo and lopap are marked with hash symbols.

The sequence of the BBPLo cDNA places it in the lipocalin protein family which is characterized by an eight-stranded antiparallel β-barrel structure with a hydrophobic central binding cavity (Fig. 2). BBPLo is 50% identical to the biliverdin-binding protein I (BvBPI) from *Samia cynthia ricini* and 35% identical to the bilin-binding protein (BBP) from *Pieris brassicae*, and insecticyanin from *Manduca sexta*. The heme-binding lipocalin nitrophorin 4 (NP4) from *Rhodnius prolixus* is only 13.5% identical with BBPLo. Generally low levels of amino acid sequence conservation are a feature of the lipocalin family, but do not usually correspond to large differences in structure. For example, NP4, insecticyanin, and BBP show only 15–16% sequence identity in structure-based sequence alignments, but the superimposed Cα structures of the three proteins show root-mean-squared deviations of only 1.5–1.6 Å, indicating a high degree of structural conservation [18].

**Figure 3:**
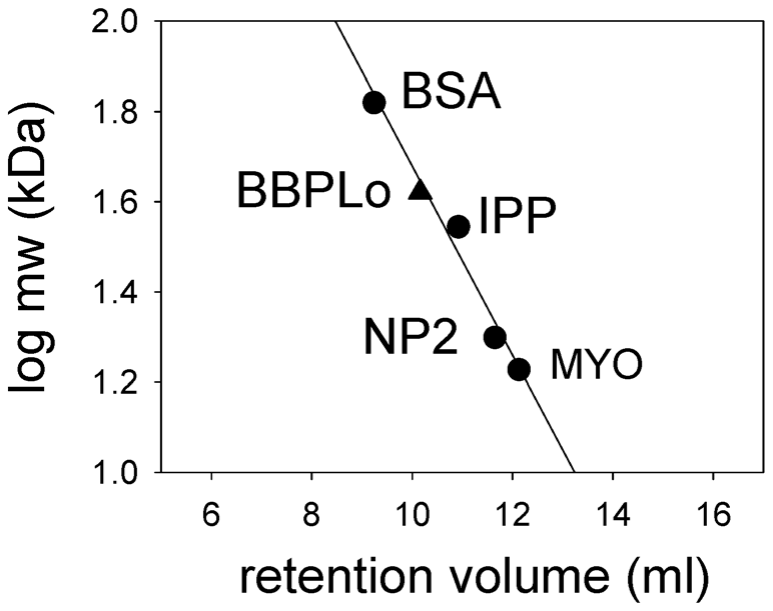
Molecular weight determination of recombinant BBPLo by gel filtration chromatography on Superdex 75. Standards: MYO (myoglobin), 16.9 kDa; NP2 (nitrophorin 2), 19.9 kDa; IPP (*R. prolixus* inositol polyphosphate 5-phosphatase), 35.0 kDa; BSA (bovine serum albumin), 66 kDa. The position of the BBPLo point is based on a calculated molecular weight of 42 kDa for a dimer of the recombinant protein.

Recombinant, refolded BBPLo was purified at a final yield of 10–20 mg/L using a combination of gel filtration and anion exchange chromatography. A molecular weight of 44 kDa was determined by analytical gel filtration chromatography (Fig. 3), suggesting that the protein occurs as a homodimer of the 21 kDa BBPLo polypeptide. Dimeric forms have also been seen with BBPs from other lepidopteran species.

### Binding of recombinant BBPLo with isomers of biliverdin

**Figure 4:**
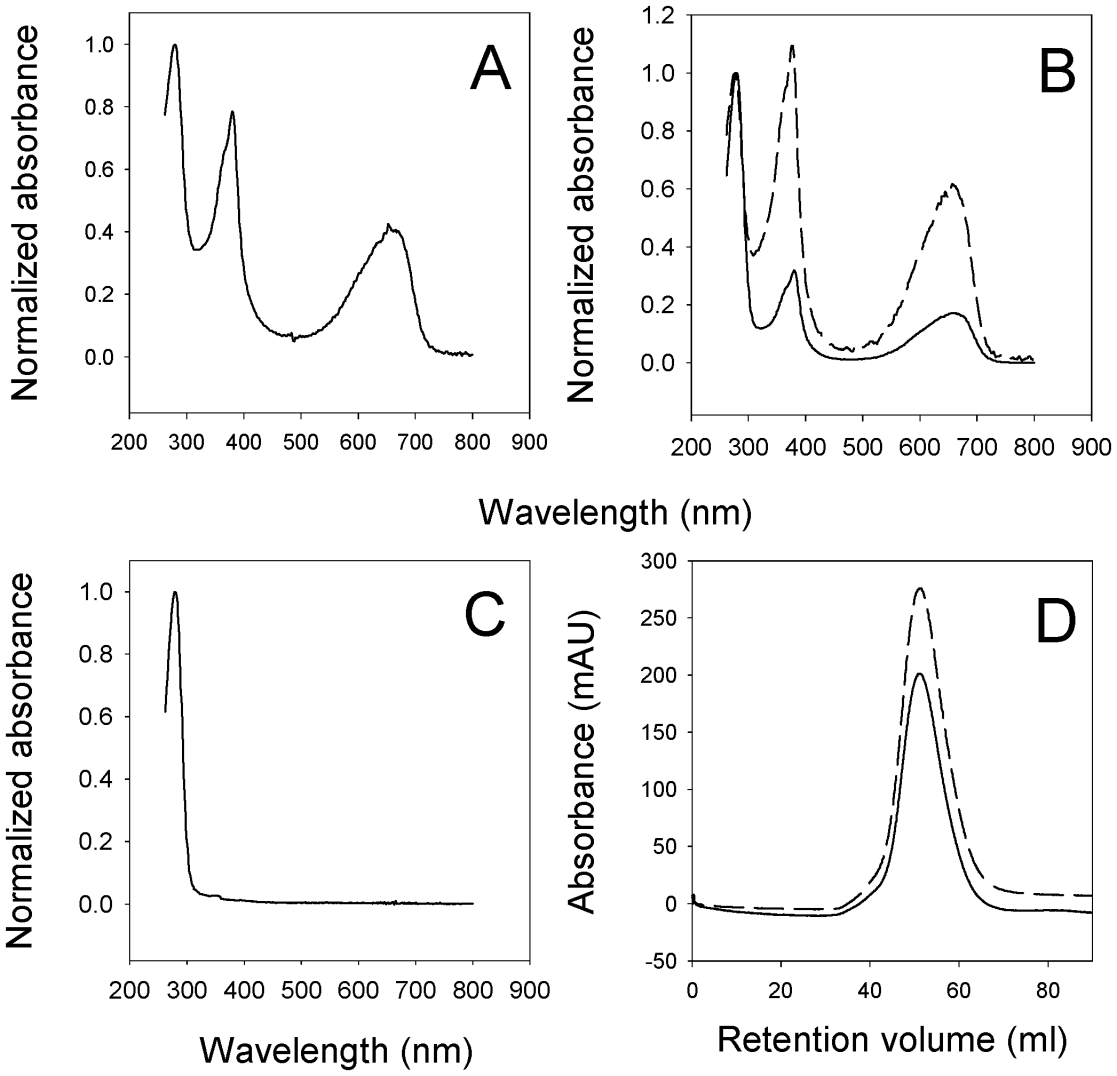
Analysis of ligand binding to BBPLo. (a) Spectral analysis of BBPLo from *L.obliqua* spicule extract using a combination of gel filtration chromatography on Superdex 75 and diode array detection. The spicule extract was injected on to the column and a full absorbance spectrum was obtained at the retention time corresponding to the elution peak of BBPLo. (b) Analysis performed as in (a) of purified recombinant protein after incubation with a sub-saturating and saturating quantities of biliverdin IXγ obtained by extraction of *M. sexta* insecticyanin. *Solid line*: Recombinant BBPLo (0.91 µM) was incubated with biliverdin IXγ (0.4 µM) prior to chromatography on Superdex 75. *Dashed line*: Recombinant BBPLo (0.91 µM) was incubated with biliverdin IXγ (1.3 µM) prior to chromatography. (c) Analysis of biliverdin IXα binding using identical methods as in panel a. (d) Heme binding to BBPLo. An excess of hemin dissolved in DMSO was incubated with recombinant BBPLo. After centrifugation the complex was purified on a Sephacryl S-100 column and absorbance was monitored at 280 (solid line) and 402 (dashed line) nm.

Samples of the α and γ isomers of biliverdin IX were added to aliquots of the recombinant protein and the mixtures were subjected to gel filtration chromatography. Biliverdin IXγ eluted with the BBPLo polypeptide and gave an absorbance spectrum similar to the BBP found in *L. obliqua* extracts and to BBPs found in other species (Fig. 4a, b) [2], [19]. Saturation of a 0.9 µM solution of BBPLo was attained with a small excess of biliverdin IXγ, suggesting that the binding constant is smaller than the protein concentration (Fig. 4b). Conversely, biliverdin IXα showed no binding with the recombinant protein (Fig. 4c). This result demonstrates that BBPLo specifically binds biliverdin IXγ making it functionally similar to BBPs previously described from the lepidopteran species *Pieris brassicae*, *Manduca sexta* and *Samia cynthia* [19], [20]. The fact that the protein forms a dimer, shows high affinity, near-stoichiometric binding of biliverdin IXγ, does not bind biliverdin IXα, and has spectral features essentially identical to the insect-derived protein, indicate that the recombinant BBPLo used in these studies was correctly folded.

### Formation of a BBPLo-heme complex

When incubated with hemin, recombinant BBPLo formed a complex which remained associated through one step of gel filtration chromatography (Fig. 4d). The spectrum of the heme complex showed a Soret maximum at 402 nm with a shoulder at approximately 385 nm. The specific content of heme as determined from the reduced pyridine hemochrome was 0.87 mole of heme per mole of protein, strongly suggesting that the complex has a 1∶1 stoichiometry. The calculated extinction coefficient for the Soret absorbance at 402 nm was 87 mM^−1^ cm^−1^

**Figure 5:**
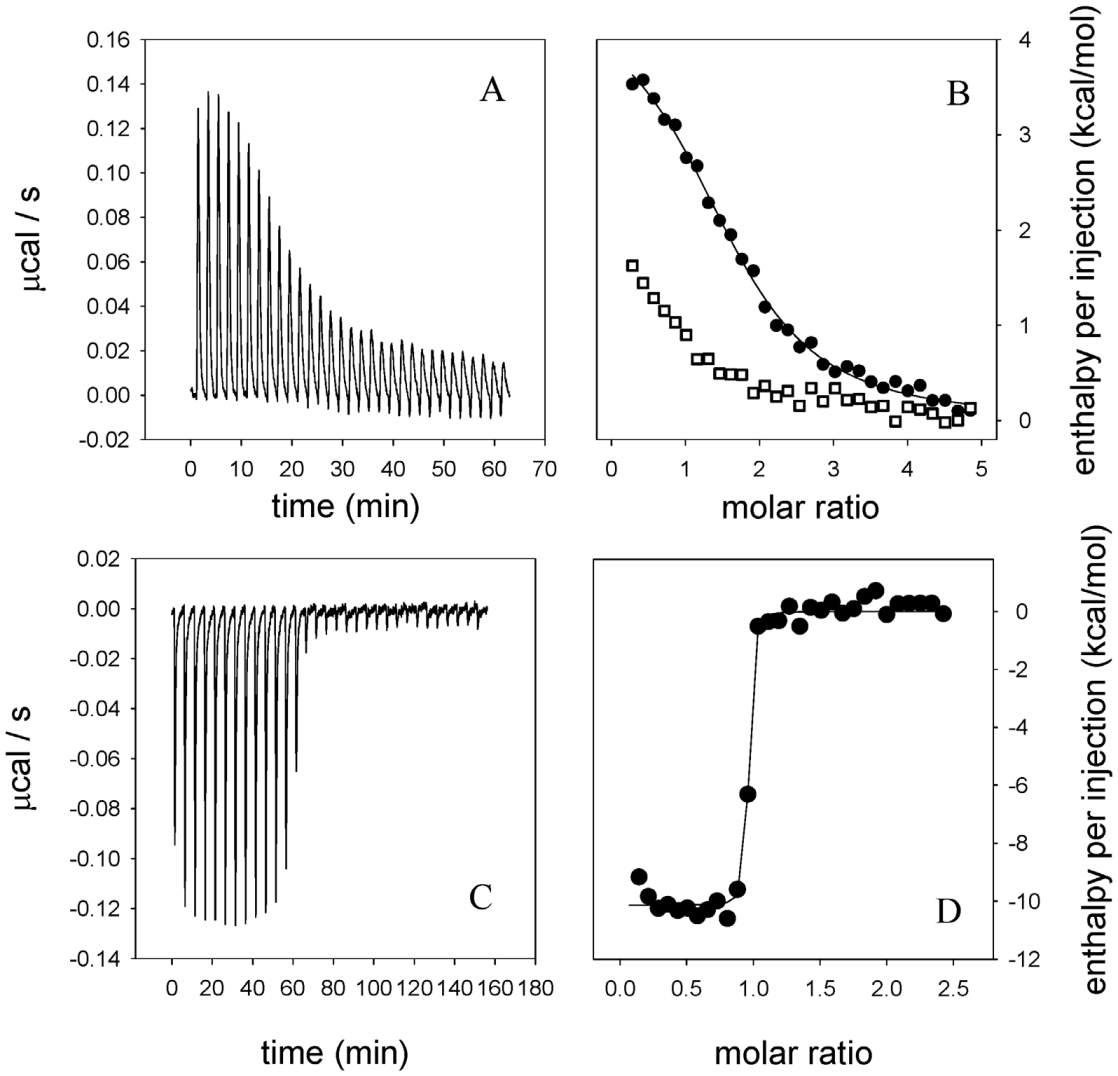
Evaluation of heme binding with BBPLo and NP2 using isothermal titration calorimetry. (a) Heats observed on injection of hemin (100 µM) into a solution of BBPLo (5 µM) under conditions described in the materials and methods. (b) Binding enthalpies plotted as a function of molar ratio for the data in panel (a) (filled circles). The line represents a fit to a single binding site model, giving the following thermodynamic parameters: ΔH = 4.5 (±0.1) kcal/mol, TΔS = 12.5 kcal/mol, K = 6.6 (±0.55)×10^5^ M^−1^. Also shown are the enthalpies (open squares) obtained when the same experiment is performed in the presence of 2 µM biliverdin IXγ. (c) Heats observed on injection of hemin (50 µM) into a solution of NP2 (5 µM) under conditions described in the materials and methods. (d) The line represents a fit to a single site binding model giving the following thermodynamic parameters: ΔH = −10.1 (±0.1) kcal/mol, TΔS = 2.3 kcal/mol, K≥9.1×10^8^ M^−1^.

The energetics of heme binding was evaluated using isothermal titration calorimetry. At 30°C the binding reaction of BBPLo with free hemin showed a relatively small positive enthalpy change and a favorable entropy change, with a calculated dissociation constant of 1.5 µM (Fig. 5a, b). The affinity is similar to that reported for heme with various heme oxygenases which range from 1–2.5 µM [15], [21]. The binding stoichiometry ranged from 1.3 to 1.7. Values in excess of 1.5 could indicate some nonspecific binding or may be due to heme aggregation as suggested by Robinson et al. [12]. When heme binding with BBPLo was performed in the presence of 2 µM biliverdin IXγ added to the cell along with the protein (5 µM), the observed heats were reduced in magnitude, suggesting that the binding sites for biliverdin and heme are coincident (Fig. 5b). For comparison, heme binding to the highly specific heme binding protein NP2 was examined. In this case the reaction showed a favorable enthalpy change, a dissociation constant of ≤1 nM and a binding stoichiometry of 0.93 (Fig. 5c, d).

### Oxidation of heme using sodium ascorbate

**Figure 6:**
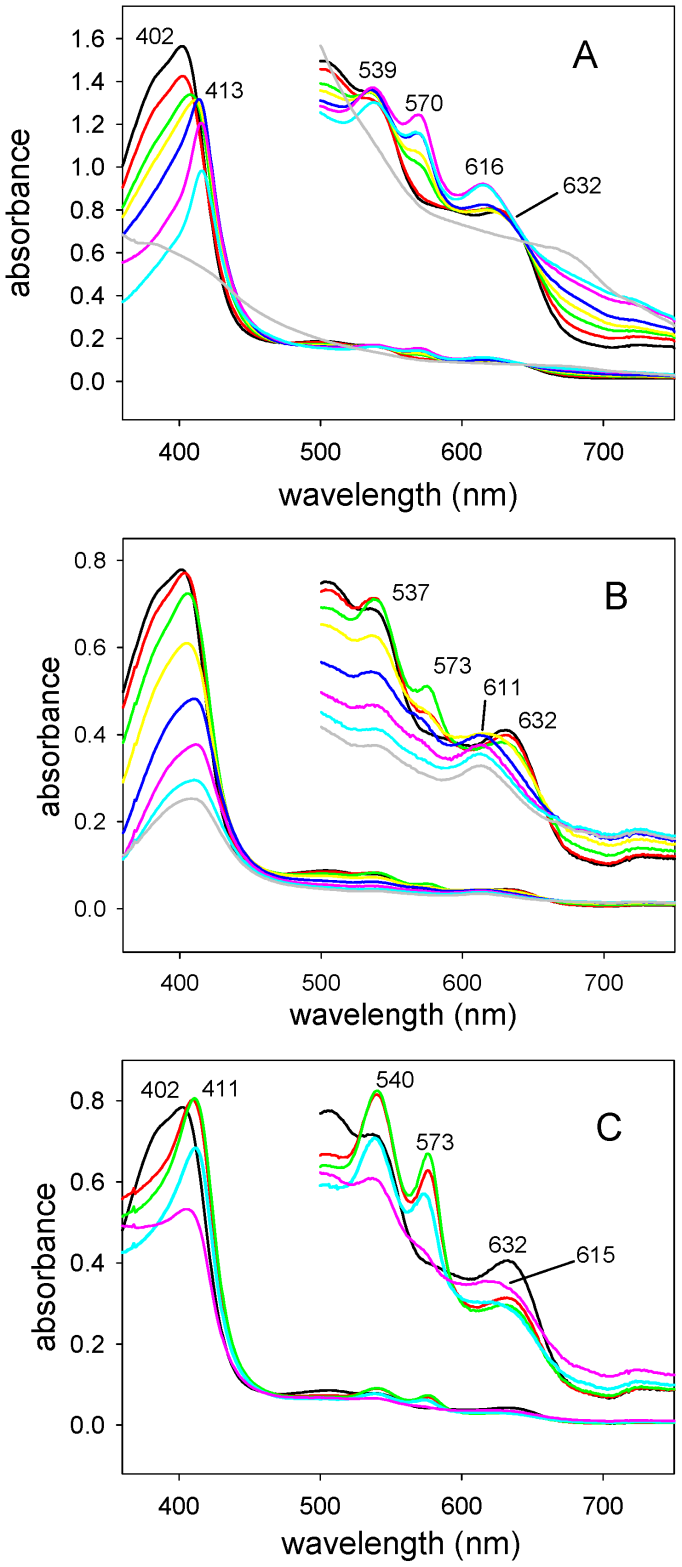
Absorbance spectra showing conversion of the BBPLo-heme complex to products. (a) Treatment of the complex with sodium ascorbate (1.4 mM) at 37°C. Spectra were recorded periodically for 3.5 hr and then after 36 hr. Labeled peaks indicate the formation of an oxyferrous complex and its conversion to a species having an absorbance maximum at 615 nm. Timepoints: Time = 0, black; 20 min, red; 40 min green; 65 min, yellow; 125 min blue; 215 min, pink; 18 hr, cyan; 48 hr, gray. (b) Reduction of the complex with an NADP^+^ -ferredoxin reductase-ferredoxin system in the absence of catalase. Timepoints: Time = 0, black; 1 min, red; 5 min, green; 15 min, yellow; 30 min, blue; 60 min, pink; 90 min, cyan; 120 min, gray. The components of the reducing system were added to both the reference and sample cuvettes, while the BBPLo heme complex was added only to the sample cuvette. The reaction was performed at 25°C and spectra were recorded periodically for 120 min then a final spectrum was recorded after 4 hours. (c) The experiment described in (b) with catalase (50 U) added to scavenge hydrogen peroxide. Timepoints: Time = 0, black; 4 min, red; 15 min, green, 90 min, cyan; 180 min, pink.

**Figure 7:**
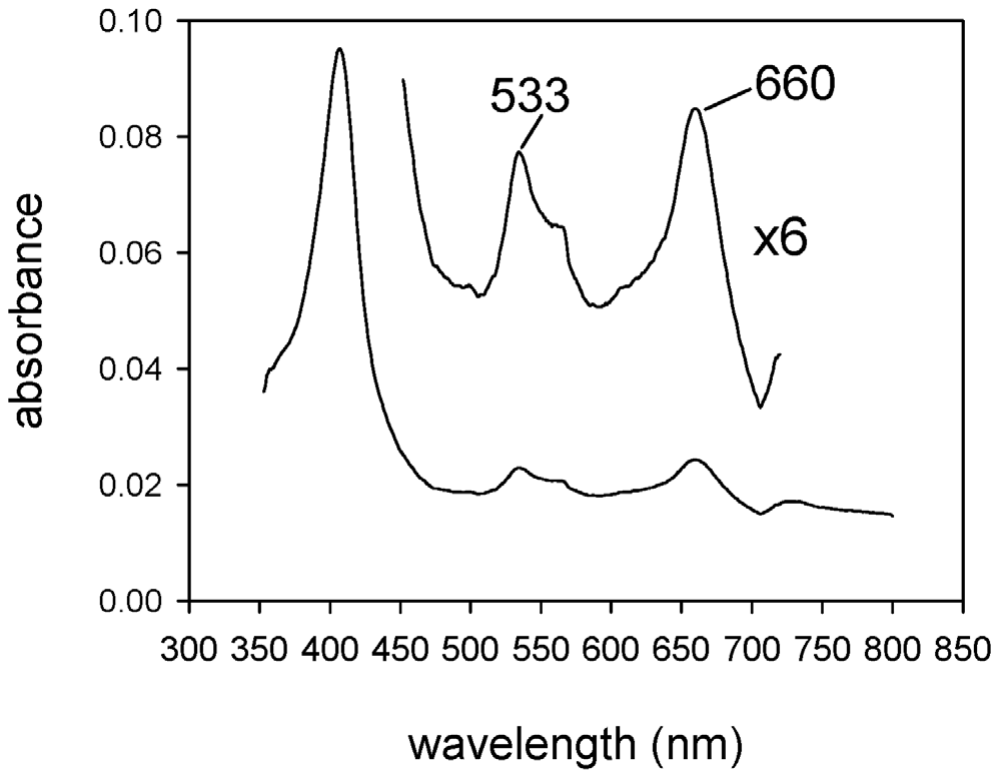
Spectral analysis of 10% pyridine in chloroform extract of reaction (as described in Fig. 6c) after 2 hr. Maxima at 660 and 533

In order to determine if bound heme could be converted to biliverdin *in situ* by coupled oxidation, the BBPLo-heme complex was incubated with 10 mM L-ascorbate. A slow shift in the Soret band to 415 nm occurred and α and β bands at 570 and 539 nm appeared suggesting the formation of an oxyferrous intermediate (Fig. 6a). Concurrent with these changes, small increases in absorbance were detected in the region between 600 and 700 nm, and a peak at 615 nm began to appear. At longer reaction times, the Soret maximum became smaller and the α and β bands began to disappear, while the peak at 615 nm became larger, and the absorbance between 600 and 700 nm also continued to increase (Fig. 6a). These spectral changes are suggestive of conversion to verdoheme or its carbonmonoxy complex (Fig. 1) [22]. This conversion was verified by extracting the reaction mixture after overnight incubation at 37°C with 10% pyridine in chloroform. The spectrum of the extract was identical to published spectra of the reduced pyridine hemochrome of verdoheme (Fig. 7).

### Oxidation of heme using a spinach NADP^+^-ferredoxin reductase - ferredoxin system

The spectral changes observed with ascorbate-mediated coupled oxidation of the BBPLo-heme complex were also seen when spinach NADP^+^-ferredoxin reductase and ferredoxin were used as a source of electrons. With the enzymatic electron transfer system, however, changes occurred more rapidly. Formation of the oxyferrous complex was observed within 5 min at 25°C, and essentially complete loss of the α and β bands with formation of a peak at 614 nm occurred within 2 h (Fig. 6b). Again, extraction with pyridine/chloroform showed the presence of the reduced verdohemochrome, but in this case the conversion was not complete.

### The effect of catalase on the reactions of the BBPLo-heme complex

Coupled oxidation of myoglobin has been found to differ mechanistically from the heme oxygenase reaction. Coupled oxidation by ascorbate depends on the presence of free hydrogen peroxide which reacts with the ferrous intermediate or the oxyferrous complex leading to the formation of verdoheme [23], [24]. Alternatively, the heme oxygenase reaction proceeds through an Fe(III)-peroxy intermediate formed by reduction of the oxyferrous complex [4]. Both reaction mechanisms lead to the formation of *meso*-hydroxyheme which reacts with oxygen to form verdoheme. To evaluate the involvement of hydrogen peroxide in the reaction of BBPLo-heme complex with ascorbate, or the NADP^+^-ferredoxin reductase-ferredoxin system, catalase (0.4 µM) was added to the reactions. With the ferredoxin-ferredoxin reductase system, rapid formation of the oxyferrous complex was observed (k_obs_ = 0.6 min^−1^). The complex was quite stable, being only partially reoxidized to the ferric form of BBPLo over a period of 5 hr. Extraction of the reaction product with 10% pyridine in chloroform showed no evidence of verdoheme formation.

With the ascorbate reaction system, the rate of spectral change was slowed in the presence of catalase, but the formation of some verdoheme was indicated by increased absorbance at 615 nm. Extraction with pyridine/chloroform yielded detectable verdoheme, but the degree of conversion was much smaller than in the absence of catalase. These results suggest that a hydrogen peroxide-mediated reaction mechanism accounts for the entire reaction product observed with the ferredoxin-ferredoxin reductase system (Fig. 6c), and nearly all of the product formed during oxidation of BBPLo with ascorbate. This suggests that bound heme bound by BBPLo is not oxidized in situ via a heme oxygenase mechanism.

### Analysis of oxidation reaction products

The reaction products formed from overnight oxidation of heme with ascorbate as an electron donor were analyzed by mass spectrometry. When the reaction mix was introduced directly, three reaction products were observed. The major product gave a peak at m/z 647, consistent with the carbonyl complex of reduced verdoheme. Also detected were smaller amounts of free verdoheme (m/z 619).

The regioselectivity of the reaction was determined after extraction of the reaction products and conversion to their dimethyl esters. A combination of reversed phase chromatography with UV detection followed by nanospray mass spectrometry was used to analyze the products. The retention times and mass spectra were compared with those of authentic biliverdin IXγ and IXα dimethyl esters.

**Figure 8:**
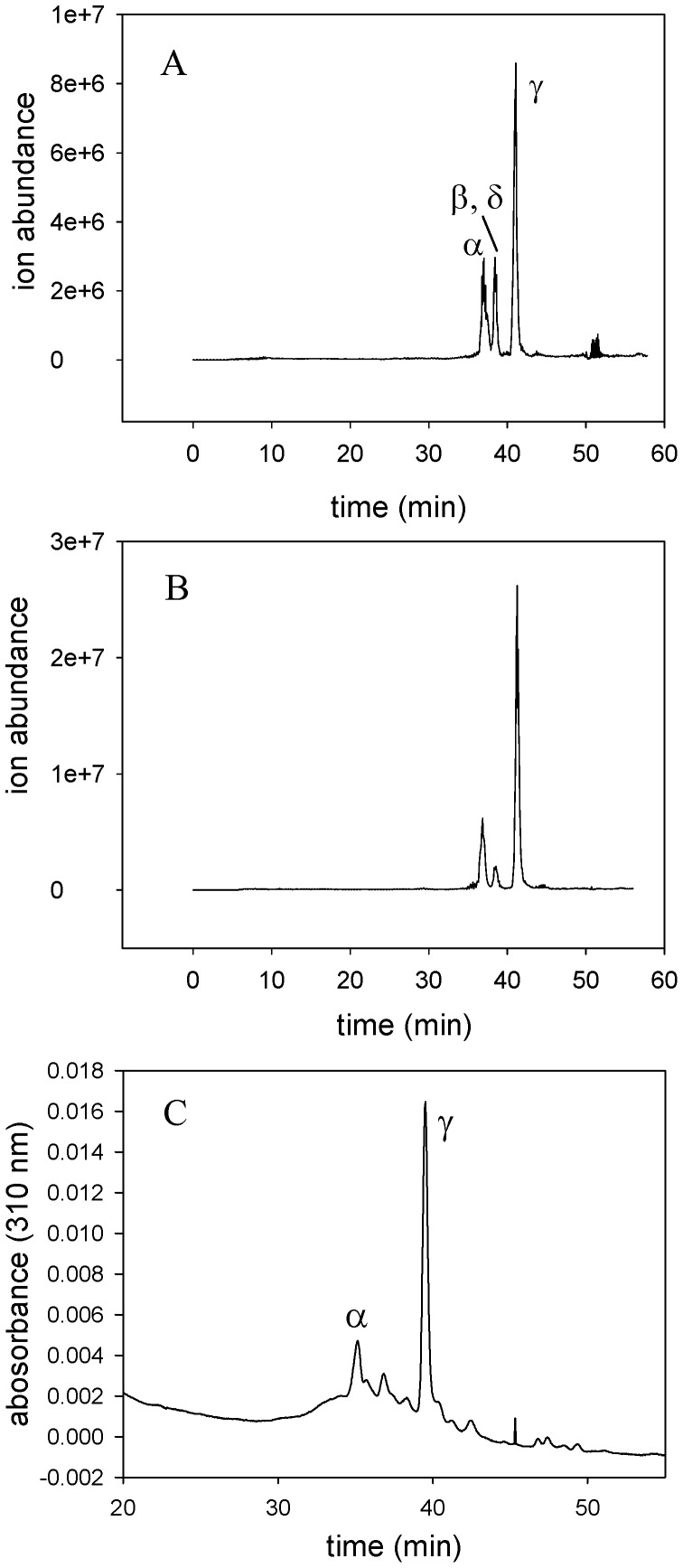
Analysis of products from reaction of the BBPLo complex with the NADP+ - ferredoxin reductase-ferredoxin system. After a four hour reaction period, the reaction was extracted and methyl esters were synthesized as described in the Materials and Methods section. The products were separated by reversed phase HPLC and detected by absorbance at 310 nm followed by nanospray mass spectrometry. (a) Selected ion trace in the mass range from m/z 609–614 showing the isomers of biliverdin methyl ester as labeled. These are the products after 1 hr of incubation in the presence of catalase, when substantial amounts of heme are still present. (b) The same mass range as (a) after 4 hr reaction. At this point, little heme remains. (c) Absorbance trace of reaction (b) monitored at 310 nm.

**Figure 9:**
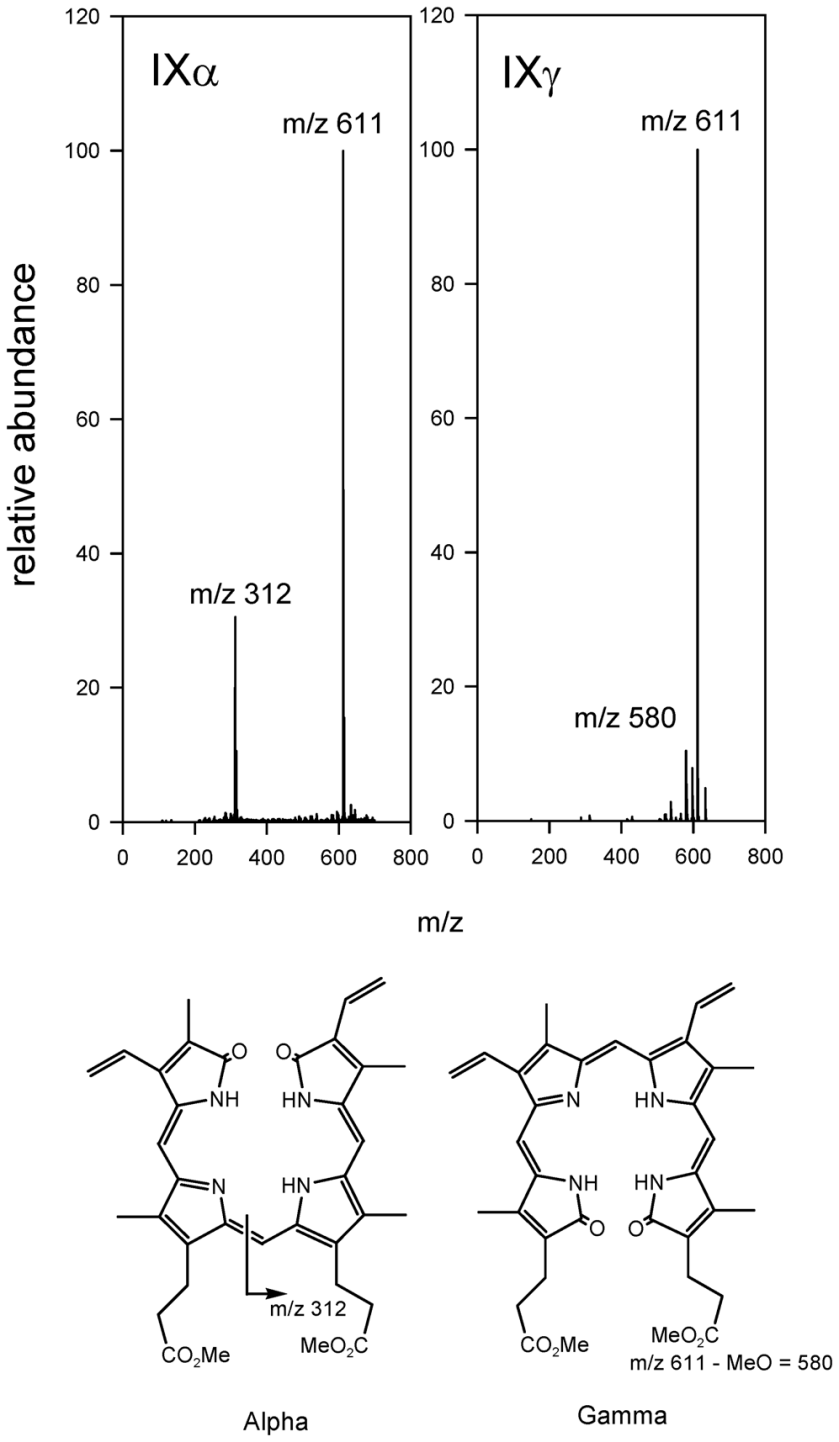
Mass spectra of dimethyl ester derivatives of reaction products. Spectra are from analysis of actual reaction mixtures and identified by comparison with those of the methyl ester derivatives of authentic biliverdin IXα and IXγ. The lower panels are structural rationalizations of the observed cleavage patterns of biliverdin IXα and IXγ methyl esters.

The major product obtained after incubation of BBPLo-heme complex with ascorbate or the NADP^+^-ferredoxin reductase - ferredoxin system co-chromatographed with the dimethyl ester of authentic biliverdin IXγ (Fig. 8). Biliverdin IXα and IXγ dimethyl esters could be distinguished by comparison of the fragmentation patterns with those of authentic standards (Fig. 9). The dimethyl esters of the major reaction product and standard biliverdin IXγ produced identical spectra having a molecular ion at m/z 611 and a fragment ion at m/z 580. The spectra of the minor reaction product and the biliverdin IXα standard again showed a molecular ion at m/z 611 but also displayed a prominent fragment ion at m/z 312, apparently representing cleavage at the γ-*meso* carbon (Fig. 9). Production of small amounts of the β and δ isomers was also suggested by the presence of a peak displaying a fragment ion at m/z 345 which eluted between the α and γ isomers. Integration of the UV absorbance trace indicated that the product ratio of the γ to α forms was 86∶14.

### Evaluation of prothrombin activation by BBPLo

**Figure 10:**
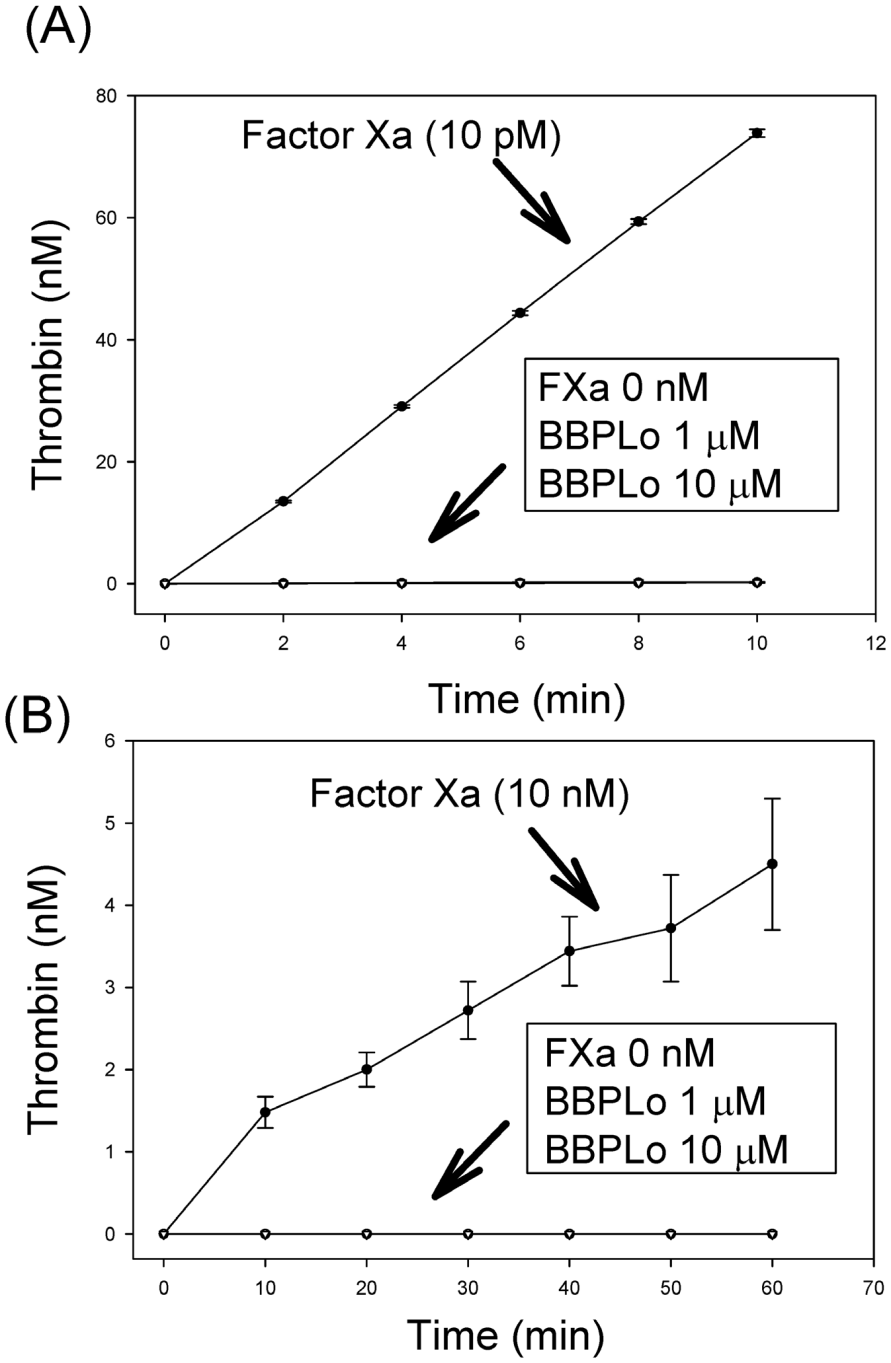
BBPLo does not substitute FXa in the prothrombinase complex (a), nor directly activates FX (b). In both panels the points represent the mean of three replicate measurements and error bars represent the standard error of the mean. (a) Prothrombin (1.4 µM) activation by FXa (10 pM) or BBPLo (1 µM) or (10 µM) in the presence of FVa (1 nM) and 10 µM PC/PS vesicles (prothrombinase complex) as described in the Methods Section. (b) Activation of prothrombin (1.4 µM) by FXa (10 nM) or BBPLo (1 µM and 10 µM) in the absence of FVa and PC/PS vesicles was also evaluated. Thrombin formation was detected using the specific substrate S-2238 (312.5 µM) and the amount of enzyme formed was calculated using a standard curve.

BBPLo has been reported to act as a prothrombin activating enzyme that plays a role in the toxicity of *Lonomia obliqua* secretions [1]. Using our recombinant preparations we compared the activity of BBPLo to that of human FXa and the reconstituted human prothrombinase complex under a variety of conditions (Fig. 10). As expected, reconstitution of the prothrombinase complex using 3∶1 PC:PS large unilamellar vesicles, human FXa and human FVa gave prothrombin production rates that were 50,000 times higher than with FXa alone (Fig. 10a). When phospholipid vesicles and FVa were present, and FXa was replaced by BBPLo, no thrombin production was observed (Fig. 10a). This was true even at BBPLo concentrations that were 1×10^6^ times higher than the standard FXa concentration of 10 pM. The result was the same in absence of phospholipids and FVa (Fig. 10b). Although BBPLo is highly similar to lopap (non-identical at four positions out of a total 185 amino acids in the mature protein (Fig. 2)) in its sequence we could not see significant activation of prothrombin as measured by hydrolysis of the chromogenic thrombin substrate S-2238 under any tested condition (Fig. 10b).

## Discussion

Based on sequence (35–50% identity) and spectral similarities with other BBPs, the BBPLo-biliverdin IXγ complex appears to be structurally similar to insecticyanin and to the *P. brassicae* BBP. Although the latter two show less than 40% amino acid sequence identity in pair-wise alignments, their superimposed crystal structures differ by only 1.2 Å in the root mean squared deviation of Cα atoms [18]. The binding mode of biliverdin IXγ in the two proteins is also remarkably similar. The ligand is arranged in the central cavity of the lipocalin β-barrel with the propionate chains projecting toward the solvent. A break in the porphyrin ring system at the γ-*meso* carbon allows it to take on a helical conformation [19], [20]. The lack of observable binding by biliverdin IXα with BBPLo is explainable by the fact that α-*meso* cleavage would not allow biliverdin to adopt this conformation. Although heme shows considerable distortion (ruffling) [25] in some proteins, it is nonetheless constrained to a roughly planar conformation. For this reason, the binding of heme with BBPLo is somewhat surprising, and suggests that the protein can adopt distinctly different conformations when binding heme or biliverdin IXγ.

### Oxidation of bound heme: products and reaction mechanism

Because of its unusual nature we hypothesized that the biliverdin ligand of the BBPs may be produced *in situ* by oxidation of bound heme rather than by a conventional heme oxygenase. To address this issue we asked if the protein-bound heme could be oxidized in the presence of electron donors, if the oxidation mechanism was consistent with the biological process of heme oxygenation, and if the products of oxidation were consistent with the exclusive formation of biliverdinIXγ.

We found that the BBPLo-heme complex can be reduced using ascorbate or an enzymatic reduction system as electron donors. The reduced BBPLo-heme complex stably binds molecular oxygen, as indicated by shifts of the Soret absorbance and the appearance of α and β bands, and the oxyferrous complex is converted to a product having an absorbance maximum at approximately 615 nm. This conversion occurs very slowly, particularly with ascorbate, requiring at least 16 hr to accumulate maximal quantities of product. The product formed in this reaction was identified as verdoheme rather than biliverdin by its absorbance spectrum after extraction with pyridine/chloroform. Mass spectral analyses indicated that verdoheme was present as its five-coordinate Fe(II)-carbonyl complex. Carbon monoxide produced in the conversion of *meso*-hydroxyheme to verdoheme is apparently not excluded from the binding pocket and forms a complex with Fe(II) verdoheme. The bound CO molecule would most likely inhibit further reaction and is probably a major factor in limiting the conversion of verdoheme to biliverdin.

Production of the biliverdin IXγ ligand by a biological process would require tight control over reaction regiospecificity. We analyzed the regiochemistry of verdoheme formation by analysis of product methyl esters using mass spectrometry and HPLC. While the results showed that reaction at the γ-carbon of heme was apparently favored, significant amounts of biliverdinIXα dimethyl ester were also detected, indicating that the reactions were not regiospecific. This suggests that the reaction of the BBPLo-heme complex with electron donors is not sufficiently regioselective to act as the source of the biological ligand biliverdinIXγ.

Verdoheme is known to be formed via two different reaction mechanisms. In the biological process of enzymatic heme oxygenation the active hydroxylating species is thought to be a Fe(III)-hydroperoxy intermediate formed by two-electron reduction of molecular oxygen. Since free hydrogen peroxide is not involved, catalase does not inhibit the reaction. Conversely, coupled oxidation of myoglobin with ascorbate involves the reaction of free hydrogen peroxide with the oxyferrous complex to produce *meso*-hydroxyheme. This reaction is inhibited by catalase. Inclusion of catalase into the reaction mixture of the BBPLo-heme complex and ascorbate greatly slowed or stopped the reaction, indicating that coupled oxidation, rather than a heme oxygenase mechanism is responsible for the formation of verdoheme from the BBPLo complex.

The fact that BBPLo is capable of binding heme, and that bound heme can be converted to a reaction intermediate on the pathway to biliverdin makes it tempting to speculate that the conversion of heme to biliverdin IXγ may occur *in situ*. However, the low overall reaction rate, the production of verdoheme rather than biliverdin, the formation of an inhibitory carbonmonoxy complex of verdoheme, the coupled oxidation-type reaction mechanism, and the lack of reaction regiospecificity suggest that biliverdinIXγ is not formed by reaction of bound heme with an unidentified electron donor. It is possible that some endogenous factor, such as a specific protein reaction partner, would facilitate the conversion of bound heme to biliverdin IXγ, but investigation of this possibility will be left for future studies.

### Tests for prothrombin activation by BBPLo

Studies by Reis et al. indicated that lopap acts as a serine protease in the activation of prothrombin [1]. In our hands, BBPLo showed no activity of this sort, when compared to the activity of the reconstituted prothrombinase complex or factor X alone. BBPLo and lopap differ at only four amino acid positions (Fig. 2). Differences this small are most likely due to allelic variation between the caterpillar populations sampled and would not be expected to affect activity of the protein. Recombinant BBPLo was eluted as a single peak from a gel filtration column and measurements of elution volume indicated a dimeric structure. BBPs are typically multimeric, and dimeric forms from other species have been described [19]. Additionally, the recombinant protein did not bind biliverdin IXα, but formed a stable complex with biliverdin IXγ. These observations provide strong evidence that recombinant BBPLo was properly folded and had a native three-dimensional structure. Further enzymatic studies with lopap itself will be needed to resolve these issues.
